# *TOP2A*, *GGH*, and *PECAM1* are associated with hematogenous, lymph node, and peritoneal recurrence in stage II/III gastric cancer patients enrolled in the ACTS-GC study

**DOI:** 10.18632/oncotarget.15895

**Published:** 2017-03-04

**Authors:** Masanori Terashima, Wataru Ichikawa, Atsushi Ochiai, Koji Kitada, Issei Kurahashi, Shinichi Sakuramoto, Hitoshi Katai, Takeshi Sano, Hiroshi Imamura, Mitsuru Sasako, for the ACTS-GC Group

**Affiliations:** ^1^ Division of Gastric Surgery, Shizuoka Cancer Center, Shimonagakubo, Nagaizumi-cho, Sunto-gun, Shizuoka, Japan; ^2^ Division of Medical Oncology, Showa University Fujigaoka Hospital, Kanagawa, Japan; ^3^ Division of Pathology, National Cancer Center Hospital East, Kashiwanoha, Kashiwa, Chiba, Japan; ^4^ Department of Surgery, National Hospital Organization Fukuyama Medical Center, Okinogami-cho, Fukuyama, Hiroshima, Japan; ^5^ Data Innovation Center, iAnalysis LLC, Minamiaoyama, Minato-ku, Tokyo, Japan; ^6^ Department of Surgery, Saitama Medical University International Medical Center, Yamane, Hidaka, Saitama, Japan; ^7^ Gastric Surgery Division, National Cancer Center Hospital, Tsukiji, Chuo, Tokyo, Japan; ^8^ Department of Gastroenterological Surgery, Cancer Institute Hospital, Japanese Foundation for Cancer Research, Ariake, Koto-Ku, Tokyo, Japan; ^9^ Department of Surgery, Toyonaka Municipal Hospital, Shibahara-cho, Toyonaka, Osaka, Japan; ^10^ Department of Surgery, Hyogo College of Medicine, Mukogawa-cho, Nishinomiya, Hyogo, Japan

**Keywords:** stomach neoplasm, DNA topoisomerase II alpha, gamma-glutamyl hydrolase, CD31

## Abstract

**Background:**

To identify factors related to relapse sites, we carried out an exploratory biomarker analysis of data from the Adjuvant Chemotherapy Trial of TS-1 for Gastric Cancer study, which is a randomized, controlled trial comparing postoperative adjuvant S-1 therapy with surgery alone in 1,059 patients with stage II/III gastric cancer.

**Patients and Methods:**

Surgical specimens from 829 patients were retrospectively examined, and 63 genes involved in a variety of biological processes were analyzed by quantitative real-time PCR. Gene expression normalized to reference genes was categorized as lower or higher than the median, and association with relapse sites was analyzed based on 5-year relapse-free survival.

**Results:**

Hematogenous, lymph node, and peritoneal recurrence developed in 72, 105, and 138 of the 829 patients, respectively; hazard ratios were 0.79 (95% confidential interval: 0.54–1.16), 0.51 (0.31–0.82), and 0.60 (0.42–0.84), respectively. Expression of platelet/endothelial cell adhesion molecule 1 (PECAM1) and topoisomerase II alpha (*TOP2A*) was strongly correlated with hematogenous recurrence and peritoneal recurrence, respectively (false discovery rate = 7.7×10^−5^ and 0.002, respectively). Gamma-glutamyl hydrolase (*GGH*) expression was moderately correlated with lymph node recurrence (false discovery rate = 0.34). Relapse-free survival was worse in patients expressing high levels of *PECAM1* (hazard ratio = 2.37, 1.65–3.41), *TOP2A* (hazard ratio = 2.35, 1.55–3.57), or *GGH* (hazard ratio = 1.87, 1.13–3.08), respectively. A multivariate analysis revealed that these were stronger independent risk factors than tumor histological type.

**Conclusion:**

In patients with stage II/III gastric cancer, *TOP2A*, *GGH*, and *PECAM1* levels in primary tumors are linked to high risk of hematogenous, lymph node, and peritoneal recurrence, respectively.

## INTRODUCTION

Despite a decreasing trend, gastric cancer remains the most common malignancy of the gastrointestinal tract in Japan and the second most common cause of cancer-related death worldwide. Gastrectomy with D2 lymphadenectomy on stage II or III gastric cancer followed by adjuvant chemotherapy with TS-1—an orally active combination of tegafur, gimeracil, and oteracil—has shown a favorable outcome. The Adjuvant Chemotherapy Trial of TS-1 for Gastric Cancer (ACTS-GC)—a prospective, randomized phase III trial of Japanese patients with stage II/III gastric cancer—revealed a 5-year survival rate of 50.2%-84.2% [1–3]. Nonetheless, some cancers recur after surgery in the form of blood-borne metastasis or peritoneal dissemination; moreover, the high variability among recurrence patterns impedes proper treatment. Hence, understanding the factors that influence recurrence can lead to the development of more effective treatments or optimization of surveillance procedures for patients with high-risk gastric cancer who undergo curative resection.

Tumor histology is correlated with the metastatic behavior of cancer cells. According to Lauren's classification, diffuse-type gastric cancer is characterized by infiltration of neoplastic cells with a significant desmoplastic response and early spreading via lymphatic dissemination, local extension into neighboring organs, or as peritoneal carcinomatosis [4]. Hepatic metastases are frequently observed in intestinal-type gastric cancer—characterized by cohesive carcinoma cells forming gland-like tubular structures with an expanding or infiltrative growth pattern—due to drainage into the portal vein. Gene expression profiles associated with cancer metastasis have been extensively studied using genome-wide approaches such as DNA microarray [5–9] and have been used to predict peritoneal relapse after curative surgery for gastric cancer [10]. An eight-gene Gastric Cancer Prognostic Score has been developed to identify patients with stage II gastric cancer who are at high risk of recurrence after surgery regardless of adjuvant treatment [11]. However, predicting metastatic risk based solely on tissue type is inadequate; furthermore, it is necessary to analyze other cohorts since previous gene profiling-based studies have been exploratory. Identifying specific genes whose expression is correlated with gastric cancer metastasis would provide biomarkers that can be used to assess the risk of recurrence.

The present study investigated gene expression in primary gastric cancer tissue in order to identify patients at a high risk for relapse at specific sites. Gene expression levels in gastric tumor tissue obtained from patients enrolled in the ACTS-GC study were evaluated by quantitative real-time PCR. In addition, we examined the relationship between the expression level of each gene and patient outcome, especially in relation to tumor relapse sites.

## RESULTS

### Demographics of the study population

Archived FFPE specimens obtained by surgical resection were available for 829 (78.3%) of the 1,059 patients enrolled in the ACTS-GC trial at 65 centers who constituted the biomarker study population. Patient demographic data and tumor characteristics (Supplementary Table 1) are summarized elsewhere [12]. The median patient age was 62 years (range: 27-80 years). As previously reported, there was no significant difference between the patients included in the current biomarker study and the total ACTS-GC trial study population.

### Gene expression

Gene expression stability measures for the reference genes *β-actin*, *glyceraldehyde 3-phosphate dehydrogenase*, and *ribosomal protein lateral stalk subunit P0* were calculated as 0.916, 0.931, and 0.923, respectively. The M values were < 1.5 for all three genes, indicating that they were suitable for target gene normalization. Four genes (*cyclin-dependent kinase inhibitor 2A*, *epidermal growth factor*, *insulin-like growth factor 2*, and *semaphorin 3B*) were excluded from further analyses because their expression levels were below the detection limit in < 60% of the samples (51.5%, 23.6%, 44.5%, and 5.5%, respectively). Thus, 59 of the 63 genes subjected to LDA met quality control criteria. The median success rate for the 59 genes was 98.6% (range: 61.2%-100%).

### Site of first relapse

**Figure 1:**
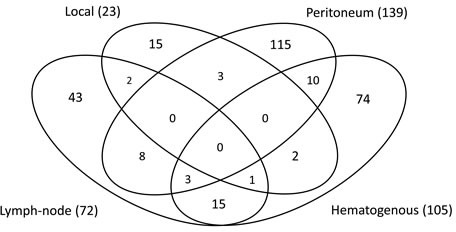
Recurrence patterns in 298 patients with documented relapse after complete resection of gastric adenocarcinoma

During the 5-year follow-up period, 291/829 patients (35.1%) experienced gastric cancer recurrence; the peritoneum, hematogenous sites, and lymph nodes were the most common sites of first relapse. Rates of metastasis and relapse were lower in the S-1 than in the surgery-only group for all sites (Table 1 and Figs. S1 and S2). Two hundred forty-seven patients (29.8%) had recurrence at a single site, 40 patients (4.8%) had recurrence at two sites, and four patients (0.5%) had recurrence at three site. (Figure 1).

**Table 1:** Site of first relapse according to treatment group*

Site	S-1 (*n* = 415)	Surgery-only (*n* = 414)	HR	95% CI
No. of relapses	123 (29.6%)	175 (42.3%)		
Local Lymph nodes Peritoneum Hematogenous	9 (2.2%) 26 (6.3%) 56 (13.5%) 49 (11.8%)	14 (3.4%) 46 (11.1%) 83 (20.0%) 56 (13.5%)	0.557 0.507 0.595 0.787	0.241–1.287 0.313–0.820 0.424–0.836 0.538–1.155

*Some patients experienced first relapse at more than one site.

CI, confidence interval; HR, hazard ratio

### Risk factors for recurrence at each site

**Figure 2:**
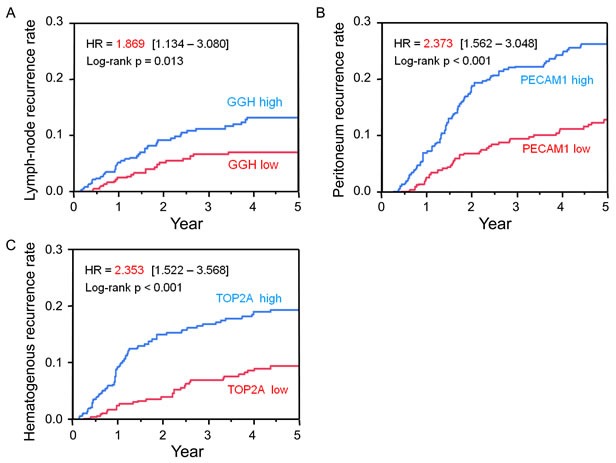
Risk factors for recurrence at each site **A.** Five-year cumulative lymph node recurrence rate for patients with *GGH*-high (blue) and *GGH*-low (red) tumors. **B.** Five-year cumulative peritoneum recurrence rate for patients with *PECAM1*-high (blue) and *PECAM1*-low (red) tumors. **C.** Five-year cumulative hematogenous recurrence rate for patients with *TOP2A*-high (blue) and *TOP2A*-low (red) tumors.

We analyzed the association between gene expression level and each recurrence site with the proportional hazard model (Table 2 and Figure 2). Among the 56 screened genes, *gamma-glutamyl hydrolase* (*GGH*) was most highly correlated with lymph node recurrence (FDR = 0.354). Lymph node relapse-free survival was lower in patients with high as compared to low *GGH* expression [hazard ratio (HR) = 1.87; 95% confidence interval (CI): 1.13-3.08]. The cumulative recurrence rate was higher in patients with high as compared to low *GGH* levels. *Platelet/endothelial cell adhesion molecule* (*PECAM*)*1* was most highly correlated with recurrence in the peritoneum [False discovery rate (FDR) = 7.7×10^−5^]. Cumulative peritoneal recurrence rate was higher in patient with high as compared to low *PECAM1* expression (HR = 2.37; 95% CI: 1.65-3.41). *Topoisomerase II alpha* (*TOP2A*) was most highly correlated with hematogenous recurrence (FDR = 0.002); the cumulative hematogenous recurrence rate was higher in patients with high as compared to low *TOP2A* levels (HR = 2.35; 95% CI: 1.55-3.57). There were no statistically significant interactions between *GGH*, *PECAM1*, or *TOP2A* expression and S-1 treatment at the respective recurrence sites, and no statistically significant factors were correlated with local recurrence (Supplementary Table 2).

**Table 2:** Risk factors for recurrence at each site

Lymph-node recurrences					Peritoneum recurrence					Hematogenous recurrence				
Gene	H.R.	95% CI	Log-Rank P	FDR	Gene	H.R.	95% CI	Log-Rank P	FDR	Gene	H.R.	95% CI	Log-Rank P	FDR
*GGH*	1.869	1.134 - 3.080	0.013	0.354	*PECAM1*	2.373	1.652 - 3.408	1.38E-06	7.72E-05	*TOP2A*	2.353	1.552 - 3.568	3.3E-05	1.8E-03
*ABCB1*	0.546	0.328 - 0.908	0.018	0.354	*ABCC1*	2.147	1.498 - 3.077	0.00002	0.00056	*ERBB2*	2.227	1.484 - 3.342	7.2E-05	2.0E-03
*UPP1*	0.569	0.353 - 0.917	0.019	0.354	*THBS1*	2.052	1.444 - 2.917	4.2E-05	7.8E-04	*GZMA*	0.445	0.286 - 0.693	0.000	0.004
*ESR1*	0.580	0.353 - 0.954	0.030	0.377	*TOP2A*	0.489	0.342 - 0.699	6.0E-05	8.5E-04	*GGH*	2.155	1.413 - 3.287	0.000	0.004
*ERBB2*	1.657	1.035 - 2.655	0.034	0.377	*CAV1*	1.998	1.406 - 2.840	8.2E-05	9.2E-04	*FAS*	0.486	0.318 - 0.744	0.001	0.008
*TYMP*	0.651	0.406 - 1.045	0.073	0.637	*GGH*	0.489	0.335 - 0.715	0.000	0.001	*ESR1*	0.512	0.337 - 0.776	0.001	0.012
*BAX*	0.674	0.421 - 1.080	0.099	0.637	*IGF1R*	1.916	1.356 - 2.708	0.000	0.001	*LRP5*	1.880	1.262 - 2.803	0.002	0.013
*TOP2A*	1.467	0.918 - 2.343	0.107	0.637	*ITGB3*	1.901	1.336 - 2.705	0.000	0.002	*VEGFA*	1.745	1.175 - 2.593	0.005	0.037
*PECAM1*	0.692	0.432 - 1.108	0.123	0.637	*EZH2*	0.518	0.360 - 0.745	0.000	0.002	*PTGS2*	0.635	0.426 - 0.948	0.025	0.144
*FAS*	0.691	0.425 - 1.122	0.133	0.637	*TYMS*	0.564	0.399 - 0.798	0.001	0.006	*EGFR*	1.571	1.053 - 2.344	0.026	0.144
*ITGB3*	0.711	0.445 - 1.135	0.151	0.637	*EGFR*	1.768	1.249 - 2.501	0.001	0.006	*VCAM1*	0.651	0.44 - 0.962	0.030	0.152
*PLA2G2A*	0.674	0.381 - 1.191	0.171	0.637	*SPARC*	1.753	1.243 - 2.472	0.001	0.006	*ABCC1*	0.658	0.44 - 0.984	0.040	0.183
*MGMT*	0.720	0.446 - 1.161	0.176	0.637	*DPYD*	1.706	1.206 - 2.414	0.002	0.010	*PLA2G2A*	0.643	0.414 - 0.998	0.047	0.183
*REG4*	0.721	0.446 - 1.165	0.180	0.637	*MAPT*	1.804	1.214 - 2.681	0.003	0.012	*ABCB1*	0.659	0.435 - 0.998	0.047	0.183
*CAV1*	0.728	0.456 - 1.163	0.182	0.637	*LDHA*	0.626	0.445 - 0.881	0.007	0.024	*IGF1R*	1.466	0.996 - 2.158	0.051	0.183
*EZH2*	1.395	0.854 - 2.278	0.182	0.637	*ESR1*	1.626	1.139 - 2.319	0.007	0.024	*PLAU*	0.681	0.459 - 1.008	0.053	0.183
*LRP5*	1.362	0.850 - 2.183	0.197	0.641	*TOP1*	0.645	0.458 - 0.908	0.011	0.036	*FPGS*	1.460	0.989 - 2.158	0.056	0.183
*VCAM1*	0.741	0.465 - 1.181	0.206	0.641	*BCL2*	1.606	1.108 - 2.329	0.012	0.036	*MUC2*	0.661	0.427 - 1.024	0.062	0.192
*GZMA*	0.742	0.460 - 1.198	0.221	0.651	*ABCB1*	1.526	1.069 - 2.178	0.019	0.056	*BCL2*	0.669	0.43 - 1.04	0.072	0.206
*ABCC1*	0.776	0.484 - 1.247	0.293	0.776	*RRM2*	0.680	0.483 - 0.957	0.026	0.073	*REG4*	0.696	0.466 - 1.04	0.076	0.206
*BCL2*	0.768	0.451 - 1.308	0.330	0.776	*VCAM1*	1.453	1.032 - 2.045	0.031	0.083	*RUNX3*	0.706	0.478 - 1.041	0.077	0.206
*APC*	0.797	0.500 - 1.268	0.337	0.776	*ERCC1*	1.401	1.001 - 1.961	0.049	0.124	*TYMP*	0.735	0.498 - 1.085	0.120	0.294
*HDAC1*	1.246	0.781 - 1.988	0.356	0.776	*TGFA*	1.405	0.993 - 1.988	0.054	0.129	*PECAM1*	0.737	0.5 - 1.086	0.121	0.294
*TOP1*	1.245	0.779 - 1.991	0.358	0.776	*E2F1*	0.704	0.491 - 1.010	0.055	0.129	*CAV1*	0.763	0.518 - 1.122	0.168	0.392
*LDHA*	1.220	0.766 - 1.942	0.402	0.776	*DAPK1*	1.377	0.974 - 1.947	0.069	0.148	*ANGPT2*	1.296	0.87 - 1.93	0.200	0.449
*ERCC1*	0.822	0.516 - 1.308	0.406	0.776	*AREG*	0.734	0.525 - 1.026	0.069	0.148	*RRM1*	1.263	0.856 - 1.863	0.238	0.509
*TGFA*	1.220	0.755 - 1.972	0.416	0.776	*UMPS*	0.730	0.515 - 1.035	0.076	0.148	*HPSE*	0.795	0.539 - 1.172	0.246	0.509
*SPARC*	0.829	0.521 - 1.320	0.429	0.776	*MUC2*	0.718	0.497 - 1.037	0.076	0.148	*APC*	0.819	0.556 - 1.204	0.309	0.597
*GADD45A*	1.232	0.717 - 2.115	0.450	0.776	*GADD45A*	1.407	0.962 - 2.058	0.077	0.148	*MAPT*	1.257	0.807 - 1.958	0.309	0.597
*EREG*	1.229	0.711 - 2.124	0.460	0.776	*VEGFA*	0.743	0.531 - 1.042	0.084	0.156	*DPYD*	0.823	0.558 - 1.214	0.324	0.606
*AREG*	0.846	0.531 - 1.348	0.481	0.776	*FAS*	1.345	0.952 - 1.899	0.091	0.162	*DHFR*	0.834	0.568 - 1.227	0.356	0.643
*PTGS2*	0.850	0.535 - 1.351	0.491	0.776	*APC*	1.334	0.952 - 1.869	0.093	0.162	*BCL2L11*	0.814	0.51 - 1.299	0.387	0.666
*FPGS*	0.850	0.533 - 1.357	0.495	0.776	*PLAU*	1.315	0.939 - 1.842	0.110	0.183	*MTHFR*	0.836	0.554 - 1.262	0.393	0.666
*IGF1R*	1.168	0.735 - 1.854	0.511	0.776	*PTGS2*	1.321	0.934 - 1.867	0.114	0.183	*MLH1*	0.847	0.567 - 1.264	0.414	0.683
*RUNX3*	0.858	0.537 - 1.372	0.522	0.776	*DUT*	0.763	0.545 - 1.069	0.114	0.183	*PTEN*	0.859	0.583 - 1.265	0.441	0.700
*MAPT*	0.845	0.499 - 1.431	0.531	0.776	*REG4*	1.308	0.929 - 1.843	0.123	0.191	*TYMS*	1.160	0.789 - 1.705	0.450	0.700
*VEGFA*	1.157	0.729 - 1.838	0.536	0.776	*BAX*	1.261	0.901 - 1.764	0.175	0.265	*HDAC1*	1.147	0.78 - 1.686	0.484	0.713
*MTHFR*	0.856	0.520 - 1.410	0.541	0.776	*LRP5*	1.232	0.880 - 1.725	0.224	0.331	*AREG*	0.873	0.596 - 1.281	0.488	0.713
*DPYD*	0.866	0.545 - 1.376	0.541	0.776	*MTHFR*	1.244	0.868 - 1.781	0.233	0.335	*EREG*	0.855	0.543 - 1.345	0.497	0.713
*DHFR*	0.872	0.549 - 1.385	0.561	0.786	*PLA2G2A*	0.790	0.520 - 1.199	0.267	0.374	*TOP1*	1.126	0.766 - 1.656	0.545	0.762
*UMPS*	1.140	0.710 - 1.829	0.587	0.795	*HDAC1*	0.847	0.607 - 1.183	0.330	0.451	*RRM2*	1.122	0.763 - 1.649	0.558	0.762
*E2F1*	1.132	0.708 - 1.810	0.605	0.795	*PTEN*	0.851	0.609 - 1.191	0.347	0.462	*TGFA*	1.114	0.748 - 1.659	0.596	0.794
*RRM2*	1.124	0.703 - 1.797	0.625	0.795	*ERBB2*	1.122	0.804 - 1.564	0.500	0.651	*SPARC*	0.907	0.618 - 1.331	0.617	0.804
*DUT*	0.891	0.561 - 1.415	0.625	0.795	*EREG*	0.877	0.589 - 1.307	0.519	0.661	*ERCC1*	1.087	0.741 - 1.594	0.669	0.852
*TYMS*	1.112	0.700 - 1.766	0.652	0.812	*DHFR*	1.108	0.792 - 1.550	0.547	0.681	*DAPK1*	0.945	0.638 - 1.399	0.778	0.957
*RRM1*	0.912	0.571 - 1.459	0.701	0.853	*MGMT*	1.088	0.773 - 1.531	0.629	0.766	*ITGB3*	0.956	0.651 - 1.405	0.819	0.957
*DAPK1*	1.086	0.675 - 1.748	0.733	0.860	*UPP1*	0.930	0.664 - 1.303	0.673	0.801	*LDHA*	1.034	0.703 - 1.522	0.865	0.957
*MUC2*	0.925	0.560 - 1.528	0.761	0.860	*RRM1*	0.939	0.670 - 1.314	0.712	0.813	*DUT*	1.033	0.703 - 1.518	0.867	0.957
*BCL2L11*	0.917	0.521 - 1.616	0.765	0.860	*MLH1*	1.065	0.751 - 1.508	0.724	0.813	*GADD45A*	0.966	0.62 - 1.506	0.879	0.957
*PLAU*	0.933	0.588 - 1.482	0.769	0.860	*HPSE*	0.941	0.670 - 1.322	0.726	0.813	*EZH2*	0.970	0.653 - 1.442	0.882	0.957
*ANGPT2*	0.933	0.571 - 1.525	0.783	0.860	*ANGPT2*	1.056	0.746 - 1.495	0.760	0.835	*MGMT*	0.973	0.661 - 1.431	0.888	0.957
*EGFR*	1.050	0.661 - 1.666	0.837	0.884	*RUNX3*	0.970	0.692 - 1.359	0.860	0.926	*E2F1*	1.028	0.693 - 1.525	0.889	0.957
*HPSE*	1.044	0.658 - 1.657	0.855	0.884	*FPGS*	0.980	0.70 - 1.372	0.908	0.960	*BAX*	0.977	0.667 - 1.433	0.906	0.957
*MLH1*	0.959	0.588 - 1.566	0.867	0.884	*TYMP*	1.009	0.721 - 1.41	0.960	0.989	*THBS1*	1.017	0.694 - 1.491	0.931	0.962
*THBS1*	1.040	0.655 - 1.651	0.868	0.884	*GZMA*	1.005	0.708 - 1.428	0.976	0.989	*UPP1*	0.987	0.672 - 1.449	0.945	0.962
*PTEN*	1.002	0.631 - 1.591	0.993	0.993	*BCL2L11*	0.997	0.632 - 1.572	0.989	0.989	*UMPS*	1.002	0.681 - 1.475	0.992	0.992

### Predictive value of biomarker analysis

Results of the multivariate analysis of *GGH*, *PECAM1*, and *TOP2A* expression in association with the pattern of recurrence are shown in Supplementary Tables 3, 4, and 5, respectively. These factors were found to be independent according to a logistic regression model, with hazard ratios that were comparable to those of tumor histological type.

### Gene expression correlations

There was a statistically significant correlation between the mRNA expression levels of *GGH*, *TOP2A*, and *PECAM1*. *TOP2A* level was positively correlated with that of *GGH* (Spearman's rank correlation coefficient: r = 0.57, *P* < 0.0001), while *PECAM1* was negatively correlated with *TOP2A* (r = −0.25, *P* < 0.0001) and *GGH* (r = −0.34, *P* < 0.0001). The correlation coefficient matrix of screened genes is shown in Supplementary Table 6.

### *GGH*, *TOP2A*, and *PECAM1* gene expression according to tumor histology

*PECAM1* mRNA levels were higher in diffuse-type than in intestinal-type gastric cancer, whereas the opposite was true for *TOP2A* and *GGH* expression (*P* < 0.0001).

### Subgroup analyses according to the disease stage

We performed subgroup analyses according to the disease stage (II or III). The results are shown in Supplementary Table 7. In the disease stage subgroup analyses, none of the genes were correlated to lymph node recurrence. *PECAM1* showed a significant correlation with peritoneal recurrence in both stage II (FDR *P* = 0.016) and III patients (FDR *P* = 0.022). In stage II patients, ATP binding cassette subfamily C member 1 and ERCC excision repair 1, endonuclease non-catalytic subunit showed a stronger correlation with peritoneal recurrence than *PECAM1* with FDR P values equal to 0.002 and 0.007, respectively. *TOP2A* expression correlated significantly with hematogenous recurrence in stage III patients but not in stage II patients. The other genes were not found to be correlated to hematogenous recurrence more strongly than *TOP2A* in the subgroup analyses according to tumor stage.

### Subgroup analyses according to the intervention

We performed subgroup analyses according to the intervention (surgery alone or S-1). The results are shown in Table S8. In the intervention subgroup analyses, none of the genes were correlated to lymph node recurrence. *PECAM1* showed a significant correlation to peritoneal recurrence in both surgery alone (FDR *P* = 0.005) and S-1 treated patients (FDR *P* = 0.031). In the surgery alone group, enhancer of zeste 2 polycomb repressive complex 2 subunit showed a stronger correlation to peritoneal recurrence than *PECAM1* with FDR P value 0.004. *TOP2A* expression correlated significantly with hematogenous recurrence in the surgery alone group but not in S-1 treated patients. Other genes were not found to be correlated to hematogenous recurrence more strongly than *TOP2A* in the surgery alone group.

No statistically significant interaction for site-specific recurrence was observed between 56 gene expressions and the treatment type (S-1 vs. Surgery alone) (Supplementary Table 9).

## DISCUSSION

In the present study, the relationship between the expression levels of 63 preselected genes and specific recurrence sites in patients with stage II/III gastric cancer was retrospectively analyzed. We found that *TOP2A*, *GGH*, and *PECAM1* levels were associated with hematogenous, lymph node, and peritoneal recurrence, respectively. In contrast, there were no genes that could explain local recurrence given the limited number of events.

*TOP2A* expression was highly correlated with hematogenous recurrence. High TOP2A expression increases the risk of hematogenous recurrence (HR=2.353), but it is also a good prognostic factor for peritoneum recurrences (HR=0.489). Topoisomerase II, which is involved in DNA synthesis and cell proliferation, is the target of antitumor drugs such as anthracyclines. There are a few reports directly linking TOP2A gene expression level and tumor hematogenous recurrence. However, topoisomerase II inhibitors such as razoxane inhibit the metastatic spread of tumors in experimental animals and possess antiangiogenic activity [13]. Correlation analysis revealed that the Pearson correlation coefficient between *TOP2A* and receptor tyrosine kinase *Erb-b2 (ERBB2)* in 832 samples was 0.403 (CI: 0.3439-0.45; *P* < 0001). *ERBB2* was the gene that was most highly correlated with hematogenous recurrence after *TOP2A* (HR = 2.23; 95% CI: 1.48-3.34; *P* < 0.0001; FDR = 0.002). Co-amplification of *ERBB2* and *TOP2A* has been reported in breast cancer cases, which has been attributed to their proximity to chromosome 17 (17q12 and 17q21.3-22, respectively) [14]. Thus, the same may be said of co-amplification of *ERBB2* and *TOP2A* in gastric cancer.

*GGH* showed the strongest correlation among the screened genes with lymph node recurrence; FDR for *GGH* was 0.345. However, tumor-stage and intervention subgroup analyses showed that *GGH* did not have any statistically significant correlation with lymph node recurrence. False positivity remains a concern for this gene. *GGH* encodes an enzyme that catalyzes the hydrolysis of (anti) folylpoly-gamma-glutamates by the removal of gamma-linked polyglutamates and regulates intracellular folate concentrations. *GGH* is reportedly a prognostic factor for tumors. In an animal model of lung cancer using tail vain injection of A549 cells in SCID mice, folate modulating metastatic potential was confirmed. A folate-rich diet increased lung colonization, increased distant metastasis to lymph nodes, and decreased overall survival [15, 16]. However, there have been no studies to date on the association between *GGH* and gastric cancer. *GGH* upregulation reduces folic acid levels in tumor cells; thus, leucovorin combination therapy may reduce the risk of lymph node metastasis. An open-label, randomized, multi-center phase III study of TAS-118 (a tegafur-gimeracil-oteracil potassium-leucovorin calcium oral formulation) plus oxaliplatin vs. S-1 plus cisplatin as a first-line therapy in patients with advanced gastric cancer is currently recruiting participants [17].

*PECAM1*, which was significantly correlated with peritoneal recurrence, encodes a protein involved in several processes relevant to the growth and spread of primary tumors, including angiogenesis, vascular permeability, and leukocyte trafficking out of the circulation [18]. PECAM1 promotes neovascularization in the peritoneum, cell migration, and metastatic progression. PECAM1 inhibition has been suggested as a therapeutic approach that targets the tumor microenvironment to suppress the end stages of metastatic progression, which has until now been a refractory clinical entity [19].

This study is the first to describe key genes that are significantly associated with recurrence at specific sites in patients with gastric cancer who have undergone 5-fluorouracil-based adjuvant chemotherapy. The understanding of molecular profiles associated with postoperative relapse risk may contribute to the development of proper preoperative monitoring procedures that make it possible to detect cancer recurrence earlier and more precisely based on gene expression profiling of resected tumor samples. Nevertheless, there were several limitations to the study. Firstly, it is a retrospective-prospective, designed, single exploratory biomarker study following randomized phase III clinical trials. The prognostic significance of the identified genes must be validated in a biomarker-based multi-institutional study of a comparable number of patients. Secondly, we screened a relatively small number of genes that were preselected based on published reports since we were unable to establish a reliable genome-wide profiling procedure on partly damaged RNA extracted from FFPE tissue samples. Thirdly, we could not evaluate protein levels by clinically feasible approaches such as immunohistochemistry because of the unavailability of specimens to validate candidate genes. Despite these limitations, our findings can contribute to the development of targeted/combination therapies for patients with gastric cancer who are at a high risk of relapse.

## MATERIALS AND METHODS

### Study population and design

Tumor tissue samples were collected from patients enrolled in the ACTS-GC study; the inclusion criteria and treatment protocol have been previously described [2]. The protocol used in the present study was approved by the ethics committee of the Japanese Gastric Cancer Association and the institutional review board of each participating hospital, and was in compliance with the Reporting Recommendations for Tumor Marker Prognostic Studies guidelines [20].

### Quantitative real-time PCR

Representative hematoxylin and eosin-stained slides from formalin-fixed, paraffin-embedded (FFPE) specimens were reviewed by a pathologist to estimate the tumor load. Sections (10 μm in thickness) were stained with nuclear Fast Red (Sigma-Aldrich, St. Louis, MO, USA) for manual microdissection of tumor tissue, which was carried out using a scalpel as previously described [8].

RNA was isolated from tumor tissue and cDNA was prepared as previously reported [21], with a minor modification to the extraction step for which we used RNeasy Mini Elute spin columns (Qiagen, Valencia, CA, USA). Expression levels of 63 genes were determined by qRT-PCR using a TaqMan array card (Life Technologies, Carlsbad, CA, USA) after TaqMan assay-based pre-amplification. Briefly, cDNA (2.5 μl) was pre-amplified using the TaqMan PreAmp Master Mix (2×) and a pool of TaqMan Gene Expression Assays (0.2×) in a 10-μl PCR reaction. The cycling conditions were 95°C for 10 min, followed by 14 cycles of 95°C for 15 s and 60°C for 4 min. The amplified cDNA sample was diluted 20 fold in Tris-EDTA buffer, and 25 μl were added to 25 μl of RNase-free water and 50 μl of 2× TaqMan Gene Expression Master Mix. The mixture was transferred to a loading port on the TaqMan low-density array (LDA), which was centrifuged twice and sealed. PCR amplification was carried out on a Prism 7900HT Sequence Detection System (Applied Biosystems, Foster City, CA, USA) under the following conditions: 50°C for 2 min and 94.5°C for10 min, followed by 40 cycles of 97°C for 30 s and 59.7°C for 1 min. The LDA included *β-actin*, *glyceraldehyde 3-phosphate dehydrogenase*, and *ribosomal protein lateral stalk subunit P0* as reference genes [22, 23]. Assay IDs used in the LDA are shown in Supplementary Table 1 hyperlinked to the DAVID website [24, 25]. The LDA includes the assays for genes encoding key molecules for growth factor signaling pathways, apoptotic signaling pathways, and DNA repair mechanisms, as well as 5-FU metabolism related genes [26, 27]. The cycle threshold value - which is inversely proportional to the amount of cDNA - was calculated. Relative mRNA levels are expressed as the ratio between the gene of interest and the geometric mean of the reference genes, providing a baseline measurement for the amount of mRNA isolated from each specimen. The expression level of each gene was categorized as low or high relative to the 50th percentile (median).

### Data processing and statistical analysis

The geNORM algorithm (MS-Excel add-on macro program; Microsoft, Redmond, WA, USA) was used to determine the stability of the reference genes as previously described [22]. The program calculated the gene stability, M, by determining the average pairwise variation between a particular reference gene and all other control genes. A normalization factor was calculated for genes with M < 1.5 based on the geometric mean of expression levels of selected genes. For quality control, only target genes for which data were obtained from > 60% of samples were used while the remainder were excluded from further analysis.

Categorical data were analyzed with the χ^2^ test. The Wilcoxon or Kruskal-Wallis test was used to assess correlations between groups. Survival curves were generated with the Kaplan-Meier product-limit method, and differences between them were evaluated with the log-rank test. Uni- and multivariate survival analyses were performed with a Cox proportional hazards model. Results were considered statistically significant at *P* < 0.05. All statistical analyses were carried out using SAS package v.9.1, JMP v.8.01 (SAS Institute, Cary, NC, USA), and MS-Excel (add-on macro program) software. The Benjamini and Hochberg false discovery rate (FDR) controlling procedure was used for multiple comparisons. Correlations between gene expression and prognosis were considered statistically significant at FDR *P* < 0.10.

## SUPPLEMENTARY FIGURES AND TABLES


