# Supplementary material for: TOP2A, GGH, and PECAM1 are associated with hematogenous, lymph node, and peritoneal recurrence in stage II/III gastric cancer patients enrolled in the ACTS-GC study
Source: Oncotarget. 2017 Mar 4;8(34):57574–82. doi: 10.18632/oncotarget.15895 (PMC5593668; doi:10.18632/oncotarget.15895)
Supplement: Supplementary file 1 [file oncotarget-08-57574-s001.pdf]

TOP2A, *GGH*, and *PECAM1* are associated with hematogenous, lymph node, and peritoneal recurrence in stage II/III gastric cancer patients enrolled in the ACTS-GC study

Supplementary Materials

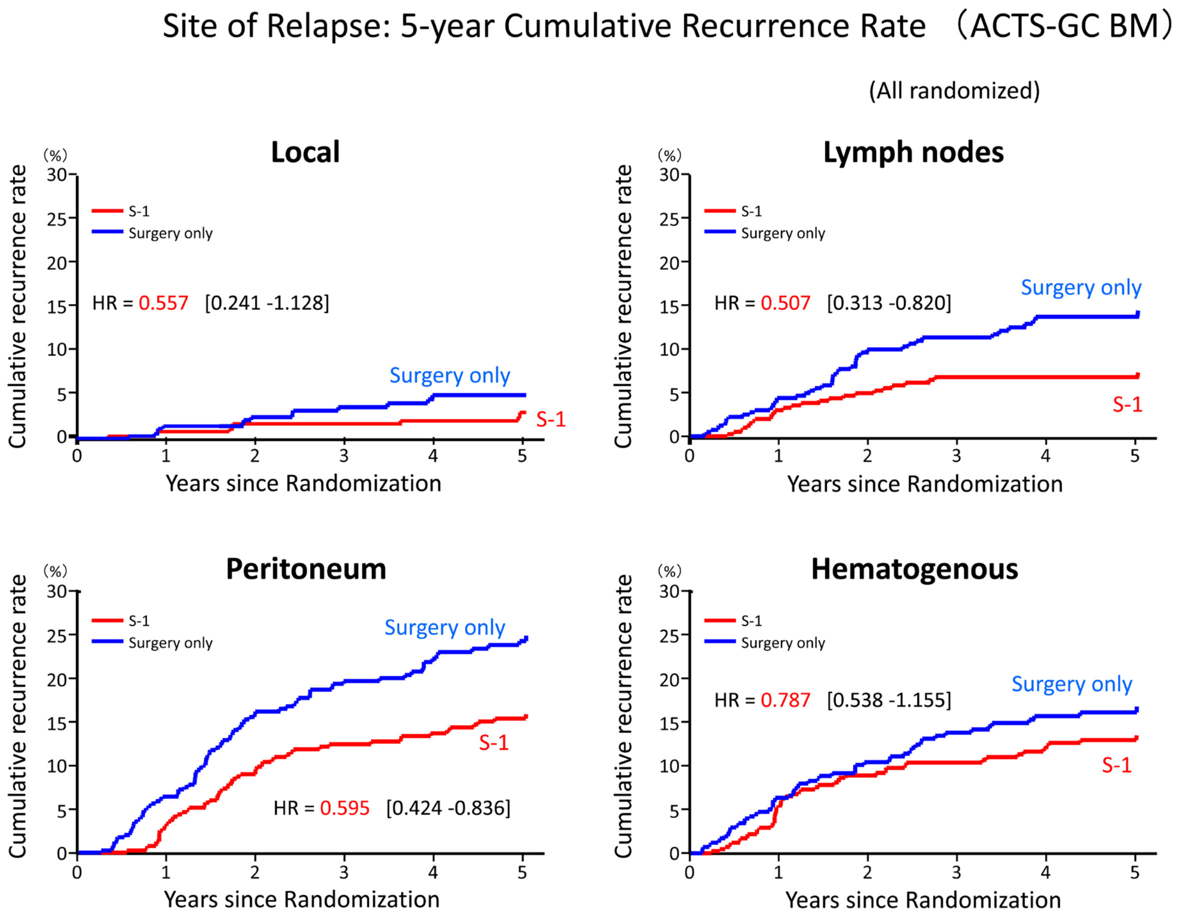

Figure S1. Site of relapse: 5-year cumulative recurrence rate.

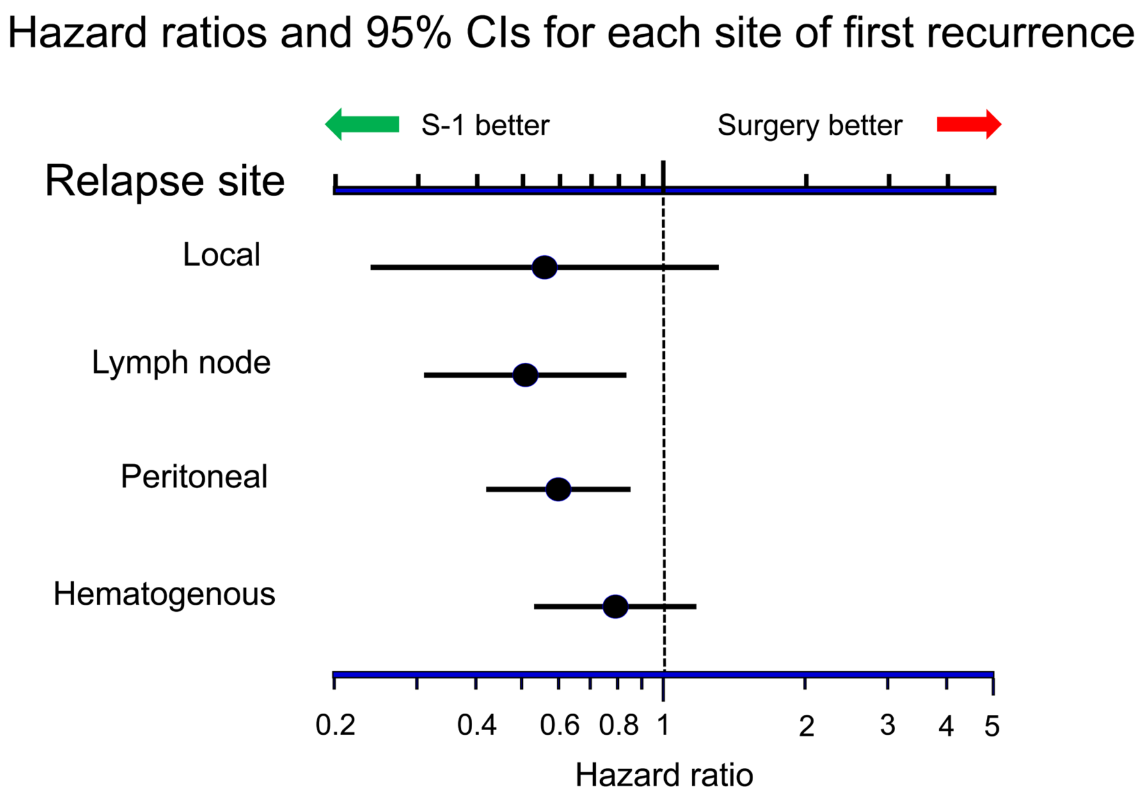

Figure S2. Hazard ratios (HR) and 95% confidence intervals (CIs) for each site of first recurrence.

Table S1: Clinicopathological characteristics of the

Clinicopathologic characteristics of the patients

	S-1 (N=415)	Surgery Only (N=414)	P Value
**Sex** - no. (%)			0.900
Male	282 (68)	283 (68)	
Female	133 (32)	131 (32)	
**Age**			0.715
<60 yr - no. (%)	160 (39)	158 (38)	
60-69 yr - no. (%)	149 (36)	161 (39)	
70-80 yr - no. (%)	106 (26)	95 (23)	
Median yr	63	62	
Range - yr	27 - 80	33 - 80	
**Tumor stage - no. (%)**			0.925
T1	1 (0)	0 (0)	
T2	222 (53)	223 (54)	
T3	180 (43)	182 (44)	
T4	12 (3)	9 (2)	
**Nodal stage - no. (%)**			0.523
N0	40 (10)	52 (13)	
N1	233 (56)	222 (54)	
N2	142 (34)	140 (34)	
N3	0 (0)	0 (0)	

.	S-1 (N=415)	Surgery Only (N=414)	P Value
**Lymph-node metastases** - no. (%)			0.175
0	40 (10)	52 (13)	
1-6	254 (61)	254 (61)	
7-15	97 (23)	85 (21)	
>16	24 (6)	23 (6)	
**Cancer stage - no. (%)**			0.480
II	183 (44)	189 (46)	
IIIa	159 (38)	162 (39)	
IIIb	73 (18)	63 (15)	
IV	0 (0)	0 (0)	
**Histologic type - no. (%)**			0.977
Differentiated	166 (40)	166 (40)	
Undifferentiated	249 (60)	248 (60)	

Cancer stage was categorized according to Japanese classification

Table S2. Univariate analysis of risk for local recurrence

Local recurrence				
**Gene**	**H.R.**	**95% CI**	**Log-Rank P**	**FDR**
E2F1	3.021	1.098 - 8.313	0.024	0.814
ITGB3	0.435	0.179 - 1.057	0.058	0.814
EREG	0.408	0.144 - 1.157	0.081	0.814
AREG	0.482	0.204 - 1.136	0.088	0.814
GZMA	0.464	0.187 - 1.151	0.090	0.814
ESR1	0.469	0.189 - 1.162	0.094	0.814
PTGS2	0.501	0.212 - 1.181	0.107	0.814
HDAC1	0.545	0.231 - 1.286	0.159	0.814
DPYD	0.547	0.232 - 1.290	0.162	0.814
MGMT	0.565	0.237 - 1.348	0.192	0.814
ABCB1	0.566	0.238 - 1.350	0.194	0.814
TYMP	0.596	0.258 - 1.377	0.220	0.814
PTEN	0.602	0.260 - 1.390	0.229	0.814
RUNX3	0.611	0.265 - 1.413	0.245	0.814
SPARC	1.634	0.707 - 3.775	0.246	0.814
BAX	0.646	0.280 - 1.493	0.303	0.814
FAS	0.648	0.277 - 1.517	0.314	0.814
BCL2L11	0.598	0.217 - 1.646	0.314	0.814
VEGFA	0.660	0.285 - 1.524	0.327	0.814
ERBB2	1.494	0.655 - 3.408	0.337	0.814
BCL2	0.653	0.267 - 1.597	0.346	0.814
UMPS	0.675	0.288 - 1.578	0.361	0.814
EGFR	0.686	0.297 - 1.586	0.375	0.814
LRP5	0.701	0.304 - 1.621	0.404	0.814
UPP1	0.717	0.314 - 1.635	0.427	0.814
TOP1	0.732	0.321 - 1.670	0.457	0.814
REG4	0.723	0.305 - 1.716	0.460	0.814
IGF1R	0.731	0.316 - 1.688	0.461	0.814
ABCC1	1.358	0.596 - 3.098	0.465	0.814
ERCC1	1.354	0.594 - 3.089	0.469	0.814
VCAM1	0.744	0.326 - 1.697	0.481	0.814
DUT	0.756	0.331 - 1.724	0.505	0.814
RRM2	0.765	0.335 - 1.745	0.524	0.814
TYMS	1.285	0.564 - 2.931	0.550	0.814
CAV1	0.785	0.344 - 1.791	0.565	0.814
PLAU	1.270	0.557 - 2.897	0.569	0.814
GADD45A	1.323	0.493 - 3.554	0.577	0.814
MLH1	0.789	0.341 - 1.826	0.579	0.814
PECAM1	0.794	0.348 - 1.812	0.584	0.814
EZH2	1.255	0.529 - 2.981	0.605	0.814
MTHFR	0.804	0.347 - 1.860	0.608	0.814
THBS1	0.814	0.357 - 1.857	0.625	0.814
TOP2A	0.814	0.357 - 1.858	0.625	0.814
MUC2	1.173	0.463 - 2.972	0.736	0.886
GGH	1.149	0.507 - 2.604	0.739	0.886
LDHA	0.886	0.391 - 2.008	0.772	0.886
FPGS	1.126	0.497 - 2.552	0.776	0.886
DHFR	0.892	0.394 - 2.021	0.784	0.886
TGFA	0.891	0.385 - 2.063	0.787	0.886
MAPT	0.893	0.386 - 2.068	0.792	0.886
APC	0.911	0.402 - 2.065	0.823	0.886
HPSE	1.071	0.473 - 2.428	0.869	0.886
PLA2G2A	1.081	0.417 - 2.801	0.873	0.886
ANGPT2	1.071	0.464 - 2.470	0.873	0.886
RRM1	0.942	0.415 - 2.134	0.885	0.886
DAPK1	0.939	0.399 - 2.211	0.886	0.886

Table S3. Multivariate analysis of peritoneal recurrence

Peritoneum recurrence

Group	Status	N	Hazard Ratio (95% CI)	P-value
Arm	Surgery Only	413	1	0.0006
	S-1	415	0.553 [0.392 - 0.776]	
SEX	Female	264	1	0.3385
	Male	564	0.843 [0.598 - 1.198]	
Stage	II	371	1	0.0000002
	IIIa	321	1.822 [1.46 - 2.274]	
	IIIb	136	3.319 [2.131 - 5.169]	
Histology	Diffrentiated	493	1	0.0038
	Undifferentiated	332	1.806 [1.204 - 2.789]	
PECAM1	Low	412	1	0.0002
	High	413	1.962 [1.367 - 2.864]	

Table S4. Multivariate analysis of lymph-node recurrence

Lymph-node recurrence

Group	Status	N	Hazard Ratio (95% CI)	P-value
Arm	Surgery Only	384	1	0.0020
	S-1	391	0.462 [0.274 - 0.757]	
SEX	Female	240	1	0.9342
	Male	535	1.024 [0.598 - 1.828]	
Stage	II	353	1	0.0002
	IIIa	296	1.868 [1.351 - 2.578]	
	IIIb	126	3.488 [1.824 - 6.644]	
Histology	Diffrentiated	460	1	0.0825
	Undifferentiated	312	0.641 [0.386 - 1.059]	
GGH	Low	386	1	0.0226
	High	386	1.803 [1.085 - 3.063]	

Table S5. Multivariate analysis of hematogenous recurrence

Hematogenous recurrence

Group	Status	N	Hazard Ratio (95% CI)	P-value
Arm	Surgery Only	413	1	0.181044
	S-1	412	0.766 [0.517 - 1.132]	
SEX	Female	261	1	0.798046
	Male	564	1.061 [0.683 - 1.694]	
Stage	II	371	1	0.000003
	IIIa	319	1.868 [1.44 - 2.421]	
	IIIb	135	3.489 [2.073 - 5.863]	
Histology	Diffrentiated	490	1	0.261429
	Undifferentiated	332	0.789 [0.521 - 1.193]	
TOP2A	Low	411	1	0.000213
	High	411	2.245 [1.455 - 3.541]	

Table S6. Correlation matrix Table (Pearson's correlation coefficient)

Table S7. Subgroup analyses according to the disease stage

Table S8. Subgroup analyses according to the intervension

Table S9. The interaction for site-specific recurrence between the gene expression and treatment arm.

Table S10. Assay IDs used in the Low-density array hyperlinked to DAVID website

For Supplementary Tables S6-S10 see Supplementary Files: Supplementary Tables S6-S10
